# The Role of Temperature in Transmission of Zoonotic Arboviruses

**DOI:** 10.3390/v11111013

**Published:** 2019-11-01

**Authors:** Alexander T. Ciota, Alexander C. Keyel

**Affiliations:** 1 Wadsworth Center, New York State Department of Health, Albany, NY 12201, USA; akeyel@albany.edu; 2 Department of Biomedical Sciences, State University of New York at Albany School of Public Health, Rensselaer, NY 12144, USA; 3 Department of Atmospheric and Environmental Sciences, University at Albany, Albany, NY 12222, USA

**Keywords:** arbovirus, temperature, vector competence, vectorial capacity, flavivirus, alphavirus, orthobunyavirus, phlebovirus, Culex, Aedes

## Abstract

We reviewed the literature on the role of temperature in transmission of zoonotic arboviruses. Vector competence is affected by both direct and indirect effects of temperature, and generally increases with increasing temperature, but results may vary by vector species, population, and viral strain. Temperature additionally has a significant influence on life history traits of vectors at both immature and adult life stages, and for important behaviors such as blood-feeding and mating. Similar to vector competence, temperature effects on life history traits can vary by species and population. Vector, host, and viral distributions are all affected by temperature, and are generally expected to change with increased temperatures predicted under climate change. Arboviruses are generally expected to shift poleward and to higher elevations under climate change, yet significant variability on fine geographic scales is likely. Temperature effects are generally unimodal, with increases in abundance up to an optimum, and then decreases at high temperatures. Improved vector distribution information could facilitate future distribution modeling. A wide variety of approaches have been used to model viral distributions, although most research has focused on the West Nile virus. Direct temperature effects are frequently observed, as are indirect effects, such as through droughts, where temperature interacts with rainfall. Thermal biology approaches hold much promise for syntheses across viruses, vectors, and hosts, yet future studies must consider the specificity of interactions and the dynamic nature of evolving biological systems.

## 1. Introduction

Global temperatures have increased by an average of 0.85 °C (0.65–1.06) from 1880–2012 [1]. The International Panel on Climate Change predicts a further acceleration of global temperature rise, with an additional 1.4–3.1 °C (RCP 6.0) or 2.6–4.8 °C (RCP 8.5) by the end of the century if there is no change to greenhouse gas emissions [1]. Temperature has been shown to have a significant influence on the transmission of many infectious agents, including arthropod-borne viruses (arboviruses) [2,3]. This results from a range of effects of temperature on biological processes influencing host, vector and virus. The complexity of enzootic transmission cycles makes understanding the role of temperature a challenge. Here, we review the role of temperature in major zoonotic arboviruses (Table 1). Viruses were limited to those that (1) are associated with human disease, (2) have a primarily non-human amplifying host, (3) are primarily vectored by mosquitoes, and (4) have at least one study examining temperature effects. We summarize studies examining temperature effects on Vector Competence, Life History Traits, Blood-feeding Behavior, Vector and Host Distribution, and Viral Distribution; and subsequently address gaps in the field that should be the focus of future studies. Since the epidemiology and ecology of each virus has been adequately reviewed elsewhere (Table 1) we focus exclusively on the influence of temperature on transmission dynamics. 

## 2. Vector Competence

Vector competence refers to the inherent capacity of an invertebrate host to become infected and ultimately transmit a given pathogen. For mosquito-borne viruses, this requires infection of the epithelial cells of the mosquito midgut following blood meal acquisition and digestion, efficient replication of the pathogen in the gut, traversing of the basal lamina of the midgut to enter the hemocoel, infection of/replication in the salivary glands, and sufficient accumulation of infectious particles in saliva for transmission to competent hosts [28,29]. Numerous studies have documented highly significant effects of adult holding temperature on vector competence for zoonotic arboviruses of interest (Table 2; [30,31,32,33,34,35,36,37,38,39,40,41,42,43,44,45,46,47,48,49]). Increases in environmental temperature increase viral replication rates in ectothermic hosts, and because viral dissemination is directly correlated to viral load [29,34,50,51], temperature increases should generally result in shorter extrinsic incubation periods (EIPs) and increased overall transmissibility. While this is largely the case, the magnitude of this effect is variable and dependent on the virus species, virus strain, dose, mosquito species, and mosquito population (Table 2; [30,31,37,44]). Kilpatrick et al. [37] demonstrated an accelerated EIP for the WNV02 genotype strains relative to NY99 genotype strains which was magnified at higher temperatures, yet similar studies by Danforth et al. [31] with distinct mosquito populations and strains measured no strain-specific effect of temperature. Additionally, while replication and subsequently EIP is generally accelerated, the effect on susceptibility is more variable and similarly species-dependent. For instance, studies with Rift Valley fever virus (RVFV) found increased infectivity at increased temperatures for *Cx. pipiens* [34] but no difference in infectivity at higher temperatures in *Ae. fowleri* [35]. There is also likely a thermal limit to the correlation between competence and temperature that is virus and mosquito-specific and independent from effects on mosquito fitness (addressed below). For instance, Vogels et al. found increased competence from 18 to 23 °C in Dutch and Italian populations of *Cx. pipiens*, yet further increases from 23 to 28 °C only increased competence in Italian *Cx. pipiens* [32]. While there are limited studies that demonstrate decreased competence above a thermal limit with zoonotic arboviruses [33,45,46], this is likely a result of experimental design (most studies have utilized maximum temperatures of between 28–32 °C). Although this review does not focus on arboviruses which utilize humans as amplifying hosts, more extensive studies assessing thermal limits and the role of interactions between mosquito genotype, viral genotype and temperature have been completed with dengue, chikungunya and Zika viruses [52,53,54,55,56,57,58,59]. A recent study by Tesla et al. which studied Zika virus competence at a temperature range from 16.0–38.0 °C found that competence was maximized at 30.6 °C with significant declines above 34.0°C, and EIP accelerated up to 36.4 °C, followed by a steep decline [54]. These relationships are likely to be highly variable with different virus strains and mosquito populations. In fact, the influence of mosquito genetics could at times supersede the generic effects of rising temperatures, even at relatively low temperatures, as has been shown by increased transmission of dengue and chikungunya at lower temperatures in some mosquito populations [53,55,56]. Given the specificity of these interactions, future studies with enzootic arbovirus should focus on establishing the relationship of temperature to competence in individual populations with circulating viral genotypes. In addition, a more thorough understanding of the mechanistic basis for population and strain-specific interactions with temperature could significantly increase our capacity to utilize genetic surveillance to predict regional impacts of climate change on arbovirus transmission.

While previous studies have focused primarily on the direct impact of temperature on viral replication and competence in adult mosquitoes, there may also be indirect effects on vector competence resulting from variable aquatic temperatures during mosquito development. In general, effects of larval temperature are more apparent in *Aedes* spp. than *Culex* spp. Studies with Venezuelan equine encephalitis virus (VEEV), RVFV, chikungunya virus and Ross River virus (RRV) demonstrated an inverse relationship between larval rearing temperature and infectivity in *Aedes* spp. mosquitoes [36,45,60], yet no effect was measured for RVFV in *Cx. pipiens* [49] Murray valley encephalitis virus (MVEV) in *Cx. annulirostris* [61] or West Nile virus (WNV) in *Cx. tarsalis* [62]. These relationships could be further complicated by additional environmental factors and larval density [63,64,65]. As temperature alters development time and mosquito size [62,66,67,68], and size has at times been associated with altered vector competence [69] this could be one important effect of temperature on competence, yet recent studies demonstrate that larval temperature can also significantly alter stress and immune gene expression [70], including influencing important proteins in the RNA interference pathway that can directly alter susceptibility in adult mosquitoes [71]. Additional mechanistic studies are needed to further probe how species and population-specific variability influences the impact of aquatic temperatures and other environmental factors on vector competence.

## 3. Life History Traits and Blood Feeding Behavior

Although experimental studies assessing the potential impact of temperature change on pathogen transmission have generally focused on vector competence, effects on development rates, longevity, gonotrophic cycle and blood feeding behavior are likely to have a larger influence on patterns and intensity of population-level transmission. 

Increased aquatic temperatures accelerate immature development [62,66,68,72,73,74,75,76,77,78,79], yet for vectors of enzootic arboviruses this effect is generally greater at lower temperature ranges (from 16.0 °C–25.0 °C), as compared to increases over 25.0 °C [62,66,72], and may be highly dependent on temperature fluctuation in addition to mean temperatures [59,72,80]. Although increased development rates in isolation would increase population size, this could be offset to some extent by increased immature and/or adult mortality, as well as decreased blood feeding, mating success, clutch size or hatch rates [66,72]. Ciota et al. found increased larval mortality for *Culex restuans* at higher temperatures (24.0 °C–32.0 °C), but no effect of temperature on immature survival with field populations of *Cx. pipiens* or *Cx. quinquefasciatus* [66]. Conversely, Grech et al. found a positive correlation between temperature and immature survival of *Cx. quinquefasciatus* from 16.6 °C to 25.2 °C [75], and a study with Egyptian *Cx. pipiens* found decreased survival at simulated mean summer-autumn temperatures (mean 30.2 °C) relative to winter-spring temperatures (mean 20.5 °C) [81]. Others have found a unimodal effect of temperature, with optimal immature survival of *Cx. quinquefasciatus* measured at 23.0 °C [73], *Cx. pipiens* form molestus at 25.0 °C [72] and *Cx. eduardoi* at 28.1 °C [78]. 

Studies of adult longevity and temperature are generally in better agreement, with decreased longevity at higher temperatures in ranges from 15.0 °C to 32.0 °C [72,73,81,82,83] and thermal thresholds for *Culex* survival generally estimated at ~34.0 °C [72,78]. The effect of temperature on the *Aedes* species may be less pronounced, although studies with enzootic vectors such as *Ae. triseriatus* and *Ae. japonicus* are limited [84,85]. As with vector competence, studies with *Ae. albopictus* and *Ae. aegypti* are more numerous and have generally demonstrated unimodal relationships for temperature and longevity, with higher optimal temperatures and thermal thresholds than enzootic vectors which generally originate from more temperate areas [54,86,87]. 

Blood feeding frequency, on a population level, should increase with development time and temperature, yet this may not always be the case if mating and/or host seeking success is negatively influenced by rising temperatures. Studies with *Cx. pipiens* and *Cx. quinquefasciatus* found an increased likelihood of blood feeding with increased temperatures from 10 °C to 28 °C [88,89], yet feeding rates could decrease at higher temperatures [66]. Differences at lower temperatures are additionally influenced by photoperiod, demonstrating that multiple environmental cues influence diapause in some mosquitoes that overwinter as adults [89]. No effect of temperature on blood feeding probability of *Ae. triseriatus* from 25.2 °C to 32.2 °C was found [85], yet extreme temperatures (<20.0 °C and >35.0 °C) have been also been shown to decrease mating [90], and could therefore negatively impact feeding frequencies.

As with vector competence, effects of temperature on life-history traits have been found to be both species and population-dependent [66,83,88,91,92,93]. The variability in experimental results demonstrates that making broad conclusions regarding the potential effect of rising temperatures on vectorial capacity is difficult. One general trend that is apparent is that optimal temperatures for vector fitness (i.e., temperatures at which population size is maximized) are generally lower than optimal temperatures for pathogens (temperatures at which competence and extrinsic incubation rates are maximized). Studies modeling transmissibility need to consider the differential effects of temperature on all aspects of vectorial capacity. There are additional caveats to these experimental studies that need to be considered. First, experimental studies, even those that have utilized fluctuating temperatures, still fail to account for the range of fluctuation of temperature and numerous other potentially critical dynamic environmental factors. Additionally, a number of studies have demonstrated that enzootic arboviruses including WNV, Eastern equine encephalitis virus (EEEV) and La Crosse Virus (LACV) can have significant impacts on mosquito longevity [50,94,95,96], fecundity [97] and blood feeding behavior [98]. Whether or not such effects are variable at different temperatures or with different viral strains has not been well studied but is suggested [83,96]. Lastly, the extensive variability on both the individual and population level demonstrates that significant plasticity exists in response to changing temperatures that likely has a genetic basis. This suggests that studies with current populations may be inept representations of future populations, which theoretically could acquire increased fitness at higher temperatures as a result of adaptive evolution.

## 4. Vector and Host Distributions

Temperature influences the distribution of vectors [99,100] and hosts [101,102] (Table 1). Mosquito vector distribution information is available for species within the USA [103,104], Europe [105], and globally [106,107]. Host distribution information is also available [108,109,110]. Birds are major hosts for many of the arboviruses (Table 1) and the distribution of avian hosts is very well understood in the USA [111,112,113] and relatively so globally [113].

Temperature effects on mosquitoes are generally thought to be unimodal [3], although the location of the mode varies by species [114,115]. For example, *Cx annulirostris*, a major vector for RRV, had increasing abundance with increasing temperature up to 32 °C (minimum ~10 °C), while *Cx. australis*, another prevalent mosquito species in the same area, had peak abundance at lower temperatures (min 6 °C), but peak abundances were lower than for *Cx. annulirostris* [114]. Similarly, while increased temperatures have been shown to increase *Cx. pipiens*, in some of the same regions, they have been shown to decrease populations of *Cx. restuans*, involved in early-season amplification of WNV [115]. Vector distribution can also lead to non-intuitive patterns of disease outbreak. For example, seasonal activity of RRV in Australia for a given vector species is later as one progresses from north to south. However, seasonal activity begins earlier in some southern areas due to the presence of a vector species that is active at lower temperatures [116]. Therefore, the direction of temperature effects vary by species and by the specific study site within a species range (e.g., [116,117]).

Consequently, in epizootic areas and endemic areas on the low side of the temperature range for arboviruses, an increase in temperature has often been associated with an increase in major vector populations. For example, increased temperatures have been associated with increased abundance of *Cx. pipiens* [115,118,119,120,121], a major vector for WNV. Increased temperatures have also been associated with an extended *Cx. pipiens* season [121]. The northern limit of EEEV is likely temperature limited due to lack of availability of suitable overwintering sites. *Cs. melanura*, the main vector for EEEV, is active at low temperatures (1–5 °C) [122], but requires liquid water in order to overwinter as larvae (typically in aquatic habitat underneath trees) [123]. In Botswana, seasonal temperature was important for *Cx. pipens* dynamics [124].

In regions with higher average temperatures, no effect of temperature variation may be detected, or effects may be mixed. For example, in Senegal, three major RVFV vector species increased in abundance with increasing temperature [125], but increased temperatures were associated with decreased RVFV hotspot risk [126]. *Cx. poicilipes* in this region was also found to increase with increased minimum temperature but decreased with increased maximum temperature [127]. *Cx pipiens* distribution in the Middle East and North Africa was primarily driven by human population density and land cover, but not by temperature or rainfall [128], although a local study in Saudi Arabia found temperature was an important predictor for RVFV vector *Cx. tritaeniorhynchus* [129]. 

Vector distributions have been explicitly modeled through species distribution modeling (SDM) (Reviewed in [130] and Table S2 of [3]). The geographic scope of these models is highly variable, with the global distribution of *Ae. aegypti* and *Ae. albopictus* having been repeatedly studied [107,131,132,133,134,135,136,137,138,139,140]. *Cx. quinquefasciatus* has also been studied globally [132,141]. Other species have global distributions (e.g., *Cx. pipiens* [142]), but to our knowledge have only been modeled within continents [143]. The capacity of vectors to adapt to local conditions have also been evaluated using a species distribution modeling (SDM) approach. The accuracy of SDMs has been evaluated using *Ae. albopictus* distribution for each continent to predict its distribution on other continents [140]. Only moderate matches between the predicted and actual distribution were found, suggesting that SDMs do not capture all factors of relevance, which is consistent with critiques of these approaches [144]. Mathematical models have also been used to model vector population dynamics [130].

Both vector and host ranges are forecast to shift further under future climate change. *Cx. pipiens*, a major vector for WNV, is already present in new locations [145], and is projected to expand its range further in Canada, especially under a high-greenhouse-gas emission scenario [143]. The distribution of *Cx. quinquefasciatus* in California and Florida is predicted to decline in mid-to-late summer, but may have increased populations during the winter [146], and range shifts are also anticipated under climate change [141]. Broad analyses indicating expected range shifts due to climate change have been performed [147]. 

## 5. Viral Distribution

A wide variety of models have been used to model temperature effects on arboviruses, including Machine Learning techniques [148,149,150], a real-time Bayesian Ensemble Adjustment Kalman Filter method [151], spatiotemporal Bayesian models [152,153], generalized linear models [150,154,155,156,157], case-crossover approaches [158], seasonal autoregressive models [159,160], R_0_ models [3], and Susceptible-Infectious-Recovered (SIR) and Susceptible-Exposed-Infectious-Recovered (SEIR) models [3,124,151,161,162,163,164,165,166,167]. West Nile virus is by far the most studied enzootic arbovirus (Table 3). Models have been reviewed for WNV [15,149,163,168], Japanese encephalitis virus (JEV) [15,16,20], RVFV [14,15], RRV [7], and Barmah forest virus (BFV) [7,169,170]. Modeling studies on temperature for remaining viruses include: EEEV [3,171], LACV [13], MVEV [3], Sindbis virus (SINV) [3,172,173], St. Louis encephalitis virus (SLEV) [3,174], and Western equine encephalitis virus (WEEV) ([174,175,176] and citations therein, [3]). Note that modeling studies that did not include temperature were omitted here (e.g., EEEV: [177,178]). The treatment of temperature in environmental models varies substantially, from simple statistical correlations to complex mechanistic models. Even mechanistic models vary in complexity. On one end, temperature based on mean climatology has been used in a simple way to correct for geographic differences [151]. At the other end, multiple life-history traits have been synthesized into a single modeling approach [3].

Despite the variety of environmental models used and the range of arboviruses examined, temperature effects have been relatively consistent. Within the main portion of the arboviruses’ range and along the cooler edge, an increase in temperature is typically associated with an increase in arboviral activity. For example, WNV infection rates north of 30° N, have been associated with increased temperatures [118,150,154,155,156,158,159,179,180,181,182,183,184,185,186,187,188,189,190,191,192,193]. An increase with increasing temperatures has often been found for JEV as well [16,194,195,196,197,198,199], as has SLEV [200,201]. WEEV was associated with increased risk with increased temperature in California’s Central Valley [176]. WEEV and SLEV have both been associated with temperatures greater than 29 °C [202]. JEV incidence increased with minimum and maximum temperatures in Jieshou county, China [203]. Temperatures above thresholds of 25.2 °C for maximum temperature and 21.0 °C for minimum temperature were associated with JEV in a temperate city in China [196]. Another study across all of China (approximately 50% of JEV cases worldwide), found JEV to increase with increasing minimum temperature, but it increased and then decreased with increasing maximum temperature [204]. Using minimum temperature, human population density, mean temperatures, and elevation, they were able to identify a high risk area with 6% of China’s land area but 60% of JEV cases in China [204]. A similar pattern has been observed south of the tropics, where there is concern that the Kunjin strain of WNV will shift further south in Australia [205,206].

Low temperatures have been found to be limiting the distribution of many arboviruses. Low temperatures are generally thought to be limiting WNV expansion northward [25,32,143,207,208,209,210,211]. Further, high temperatures have been observed to aid in the establishment of arboviruses. For example, WNV often invaded new locations following heatwaves [44], but once established no longer required such high temperatures to persist [27,212]. Indeed, simulations found that temperature was more important in WNV establishment than mosquito population composition (i.e., biotype ratio of *Cx. pipiens*), fraction of hosts that are birds, and the mosquito-to-host ratio [32]. At low temperatures (18 °C), mosquito-to-host ratio has a greater influence on WNV establishment [32]. Similarly, amplification of SLEV was estimated to stop at 17 °C [40,202] although the virus has still been detected in female mosquitoes during winter and spring when temperatures were between 11–15 °C. SLEV has also been predicted to expand further northward due to increased temperature suitability [213] as SLEV outbreaks have generally occurred at or below the 21 °C isotherm [214].

A role of temperature has also been observed within the tropics. JEV vector abundance has also been shown to increase with increasing temperature in India [215,216] but see [217] for the same study region where no significant correlation with temperature and JEV infection rate was found. Increased minimum temperature with a 6-month lag was associated with increased JEV cases in Malaysia [218]. 

However, increased temperatures may cause decreases in arbovirus activity in hotter portions of their range. For instance, high temperatures have been predicted to limit WNV in subtropical regions [117]. WEEV risk has been predicted [213] and observed [176] to decrease in the southern portion of its range with an increase in temperature. *Cx. tarsalis* is able to clear WEEV infections at 32 °C compared to 18 and 25 °C [213]. Transovarial transmission in *Cx. tarsalis* is increased for SLEV at 18 °C relative to 27 °C [219]. Data on *Cx. quinquefasciatus* suggests that WNV will decline in some portions of its Southern range with increasing temperatures [117,146]. Maximum temperature was negatively associated with seroprevalence of RVFV, although there was a strong trend towards an increase in seroprevalence with increasing night-time (minimum) temperature [220]. A large MVEV outbreak occurred during 2010/2011 due to low temperatures and high rainfall [221]. 

Within the normal range of some viruses, environmental temperatures were not found to be the main limiting factor. For example, rainfall and tides are the main environmental predictors for RRV [7] and similarly RVFV is strongly tied to rainfall [14,222,223]. Rainfall was more strongly associated with EEEV than temperature in Rhode Island, USA [171]. Rice fields, swine production, and percent of humans and swine vaccinated were more important for JEV in some regions [17]. On the island of Mayotte, where temperatures were suitable for RVFV, the import of an animal infected with RVFV was the most important risk factor [224]. No direct temperature relationships were detected for SINV in South Africa [173]. A study of seasonal and meteorological models aggregated by state in Australia found relatively small improvements of adding weather variables for RRV and BFV, with the largest improvements due to lagged variables [157]. Similarly, in Queensland, weather variables were not found to predict RRV [225].

Statistical methods can also influence whether or not temperature is significantly related to disease in a region. For example, temperature was correlated with JEV in Nepal, but was not retained in a final model when other covariates were considered (precipitation, the percentage of irrigated land, the percentage of grassland cover, and the pig-to-human ratio) [226]. Therefore, models may show a temperature effect, but the effect may disappear when another relevant covariate is included.

### 5.1. Indirect Effects

Beyond direct temperature effects, temperature can influence arboviruses indirectly through interactions with other variables. Drought has been found to be important in the amplification of Flaviviruses, with a strong association between drought and drier conditions and WNV [150,180,227,228,229,230,231] and SLEV [174,201,232,233]. Increased snow depth was predictive of increased SINV in Finland [172], suggesting an indirect role of temperature on this virus. SLEV was found to increase indirectly with low temperatures [234]: winter freezes in Florida were associated with increased avian breeding success that resulted in a larger number of susceptible hosts [235].

### 5.2. Climate Change

Many arboviruses are expected to shift poleward under climate change, due to increasing temperatures. Migratory birds with antibodies to WNV already arrive in northern Europe (Sweden) [236], demonstrating that dispersal is not limiting arboviral activity in these locations. WNV is expected to increase in Europe, even in locations where it is already present, under future climate change [209], and to increase in range and intensity in Canadian prairies under a range of future climate conditions [211]. Increased drought expected under climate change has the potential to triple WNV cases over a 30-year time frame in locations where there is low human immunity [229]. In contrast, increased rainfall has been predicted to lead to decreased WNV in some regions despite temperature increases [208]. Increasing temperatures associated with climate change may have facilitated arboviral spread up elevation gradients. For example, JEV has recently expanded into Tibet where it was formerly thought to be excluded due to high elevation [237]. RRV is expected to increase in temperate areas, but decrease in tropical areas where it is endemic due to increases in temperature [167]. In contrast, MVEV is predicted to decrease in summer and autumn in Western Australia due to higher temperatures decreasing vector survival [238]. Risk for BFV has also been assessed and was not expected to change much when both temperature and rainfall were included in the model [169].

### 5.3. Case Study: Season-Specific Effects in West Nile Virus in Temperate Regions

The role of temperature across seasons has been well-studied for WNV. While transmission season temperatures are clearly important, temperatures in the non-transmission seasons can also affect viral dynamics.

#### 5.3.1. Winter Temperatures

Warmer winter temperatures have been associated with WNV across the continental US [155,179], Russia [186,187] and in localized studies [160,191]. Low temperatures are sufficient to halt viral replication (e.g., 14.3 °C or below for WNV in *Cx. tarsalis* [44]), although infections in mosquitoes persist at these low temperatures and replication may resume in the spring [44,239]. Thus, it is expected that winter temperatures are acting on vector populations. Other modes of overwintering (e.g., within birds, reviewed in [240,241]) could provide increased capacity for seasonal maintenance that are less sensitive to temperature variation.

#### 5.3.2. Spring Temperatures

Warm and dry springs have been associated with WNV [154], especially maximum temperatures, which are likely associated with increased early amplification [149]. Temperature in May and June were linked to WNV in Russia [186]. Early-season temperatures were predictive of human cases later in the year in South Dakota [190].

#### 5.3.3. Summer Temperatures

Above average summer temperatures likely contributed to WNV epidemics in 2002–2004 [44] and hot dry summers were also identified as associated with WNV foci in Connecticut, while warm wet summers were associated with more distributed cases of WNV [242]. Minimum summer temperature was found to predict human and mosquito cases in New York and Connecticut [149]. Increased temperatures at a two-week lag interval were associated with WNV in Suffolk County, NY [152] and Nassau County, NY [153]. August and September temperatures were associated with increased WNV incidence in Russia [186]. Increased summer temperatures were associated with West Nile fever in Europe [184,189]. In Romania, temperatures twenty days earlier were found to increase WNV infection rates [227]. High temperature anomalies in July were linked to WNV outbreaks in Europe [184,243]. In contrast, in West Texas, a dry and cool summer following a wet spring was associated with increased WNV cases [244]. In locations with high summer temperatures (i.e., deserts), habitat suitability for WNV may be low during the summer [245]. In South Africa, an increase in minimum summer temperature decreased WNV infection rates [173]. Similarly, summer temperatures have also been linked to JEV in Japan [195].

#### 5.3.4. Fall Temperatures

Dropping temperatures in the fall have the potential to end the West Nile virus season. However, late season temperatures associated with the end of the WNV season have generally not been retained in final models of WNV risk [e.g., 149]. One explanation for this is that changes in host behavior and abundance are likely more important than temperature at this time of year [246]. 

### 5.4. Case Study: Barmah Forest Virus

Temperature relationships for BFV are scale-dependent, but generally risk of BFV increases with increasing temperatures. BFV was found to increase with increased minimum temperature based on 0-, 2-, 3-, 4-, and 5-month lags in Queensland, Australia (using seasonal differencing to control for seasonal effects) [247], and with increasing maximum temperature [248] (note exact attribution is difficult as minimum and maximum temperature are strongly correlated in the region, Spearman correlation coefficient of 0.93). BFV risk increased with increasing minimum temperatures in coastal regions [169]. BFV risk was decreased with increasing minimum temperature at the scale of the entire state of Queensland [170], but this effect could be due to a contrast between interior and coastal areas rather than an inconsistent temperature effect (i.e., Simpson’s paradox [249,250]).

### 5.5. Case Study: Ross River Virus

RRV is an excellent example of a virus demonstrating a temperature optimum [3], although a number of studies from different geographic regions have not reported an effect of temperature on RRV after controlling for other variables [251,252,253,254]. A study summarizing 100 years of epidemics found that relationships with temperatures varied by region, with an increase in minimum temperatures being associated with RRV in Southern Australia, an increase in RRV associated with a decrease in maximum temperatures in arid parts of Australia, and no strong relationship in tropical northern Australia, where temperatures are routinely suitable for this virus [8]. At a regional scale (defined by cluster analysis), the RRV risk decreased with increasing spring minimum temperatures in the northernmost region (closest to equator), while in the southernmost (poleward) region, RRV risk increased with increasing spring minimum and maximum temperatures, with two other regions showing no effect of temperature [225]. Similarly, in Southern Australia, RRV infections increased with either increasing monthly mean minimum or maximum temperatures [255] and temperature increased RRV risk in Tasmania [256]. Temperature was included in a model used to forecast RRV risk 1–5 weeks in advance in Western Australia [257]. Risk of RRV increased with increasing minimum temperature, except in one area (Capel, in SW Australia) [257]. Risk also generally increased with increasing maximum temperature, but decreased in Capel [257], and showed no effect in two other locations.

Temperature results in Queensland in northwest Australia have been variable, and this may be due to the stronger effects of rainfall and tides [258]. One study found that RRV incidence increased by 2.4% for each 1 °C increase (including a statistical correction for season) [259]. Temperature was not significantly related to RRV in the city of Brisbane [260] in one study, but another study found a negative effect of maximum temperature there [261]. Maximum and minimum temperature were associated with RRV risk along the coast, but not inland in another study [262]. Maximum temperature was weakly positively correlated with RRV in Townsville after accounting for other variables [263]. 

### 5.6. Case Study: Rift Valley Fever Virus

Temperature effects have also been detected in viruses where other factors have been shown to be the main determinants of viral dynamics. Rainfall and vegetation have been found to be most predictive of RVFV [264] and RVFV in south and east Africa was successfully predicted without consideration of land surface temperature [265]. However, a mechanistic model based on water temperature and surface area was used to examine a range of conditions theoretically expected to be favorable to RVFV persistence and outbreaks [164] and in Kenya, minimum temperature was among the significant variables included in a RVFV early-warning system [266]. Temperature factored into a mechanistic model for RVFV in East Africa [267]. Temperature and precipitation effects were also included in a SEIR model of RVFV in Tanzania, where an increased risk at low temperatures was found, despite an increase in overall risk under climate change ([165], but see [268] where no effect of temperature was observed). Cooler-than-normal temperatures regionally (~30 °C compared to ~40 °C) and heavy rainfall were associated with RVFV in South Africa in 2010/2011 [221], but note that spatially, epidemic outbreaks were most likely to occur in regions with temperature >32 °C, and increased risk from 25–32 °C relative to temperatures <25 °C [269]. Temperature was also included in a discriminant analysis of RVFV in South Africa [270].

Temperature has also played a strong role in risk modeling for new locations. Temperatures associated with mosquito development were used in modeling suitable locations in North Africa [271] and Spain [272] for RVFV. Specifically, a minimum of 14 °C and a maximum of 40 °C were used, with an optimum of 28–32 °C for enzootic suitability, and a linearly increasing risk for epizootic transmission. Extrinsic incubation period and gonotropic period both decreased with increasing temperature (increasing viral risk) in a model of locations suitable for RVFV in California, USA [166]. The model found that a RVFV outbreak was possible in all months except December and January. A temperature-based model for the continental USA found that the number of risk days ranged from 0 in the far north to 325 in Florida [273]. 

## 6. Concluding Remarks and Future Directions

Thermal biology is emerging as a trait-based approach to studying temperature effects, especially for arboviruses [3]. Two patterns in temperature relationships among arboviruses were revealed: in some viruses, a strong role of temperature was clear across the virus’ range, while for others, temperature was more important in setting the virus’ range, but not in governing dynamics within that range. For example, WNV frequently increased with increasing temperature, even in locations where it was endemic. In contrast, RVFV generally did not show strong temperature effects where the virus was endemic.

The relationship between temperature and viral transmission may be complex due to the variety of influences on different aspects of the ecology and biology of vectors, hosts, and viruses [3]. The fact that temperatures effects can vary at multiple time scales (i.e., within days and across seasons) can further complicate these relationships. Diurnal variations due to non-linear temperature relationships are important [57], have been observed for WNV [183], and can be accounted for in analyses of arboviruses [3]. Spatial variation in temperature is also important, and is not well represented on fine scales (i.e., microclimatic variation) by gridded temperature products [274,275]. This can lead to underestimation of vector-borne disease risk in some habitats and locations [276].

Where arboviruses are emerging, more refined spatial and temporal data could improve early warning forecasting systems. High-quality, publicly available temperature data sets exist, but vector species abundances and locations and virus infection rates need to be more publicly available in accessible Geographic Information System (GIS) formats. Improved knowledge of vector and host distribution would also be instrumental in improved forecasts of expected changes under climate change. Host-vector interactions are additionally important, and the degree to which host choice depends on temperature is also worthy of study. Host choice in *Cs. melanura*, for example, has also been found to be temperature dependent [277], however temperature co-varied with season, which needs to be controlled for in future studies. Temperature effects can vary by vector species and even within populations of a single species. Vector and host populations can evolve and adapt, and temperature will likely have significant effects on both the rate and trajectory of viral evolution. For this reason, models need to be informed by experimental studies that consider not just how changing temperatures interact with current biological systems, but also future biological systems. Further, the degree to which temperature relationships interact with other environmental variables (e.g., precipitation, insecticide resistance, vaccine development) should be considered. Defining these complex and nuanced relationships over appropriate temporal and geographic scales, while daunting, is critical if we are to accurately define how climate change will alter the transmission dynamics of mosquito-borne viruses. 

## Figures and Tables

**Table 1:** Zoonotic mosquito-borne viruses commonly associated with human disease.

Species	Primary Vector	Primary Hosts	Distribution ^1^	Human Disease
***Togaviridae: alphaviruses***				
*Eastern equine encephalitis virus (EEEV)* [4]	mosquito (*Culiseta*, *Culex*)	bird	NA, C/SA	febrile illness, encephalitis
*Western equine encephalitis virus (WEEV)* [5]	mosquito (*Culiseta*, *Culex*)	bird	NA, C/SA	febrile illness, encephalitis
*Sindbis virus (SINV)* [6]	mosquito (*Culex*)	bird	AF, EU, AS, ME, AU	febrile illness, arthralgia
*Ross River virus (RRV)* [7,8,9,10]	mosquito (*Aedes*, *Culex*)	mammals (marsupials)	AU	febrile illness, arthralgia
*Barmah forest virus (BFV)* [7]	mosquito (*Aedes*)	mammals (marsupials)	AU	febrile illness, arthralgia
*Venezuelan equine encephalitis virus (VEEV)* [11]	mosquito (*Aedes*, *Culex*)	small mammal, equids	NA, C/SA	febrile illness, encephalitis
*Mayaro virus (MAV)* [12]	mosquito (*Haemagous*)	non-human primate	C/SA	febrile illness, arthralgia
***Bunyaviridae: orthobunyaviruses***				
*Lacrosse virus (LACV)* [13]	mosquito (*Aedes*)	small mammal	NA	febrile illness, encephalitis
***Bunyaviridae: phleboviruses***				
*Rift Valley fever virus (RVFV)* [14,15]	mosquito (*Aedes*/*Culex*), phlebotomus flies	mammal (ruminants)	AF	febrile illness, hemorrhagic fever, encephalitis
***Flaviviidae: flaviviruses***				
*Japanese encephalitis virus (JEV)* [15,16,17,18,19,20]	mosquito (*Culex*)	bird, swine	AS	febrile illness, encephalitis
*Murray valley encephalitis virus (MVEV)* [21]	mosquito (*Culex*)	bird	AU	febrile illness, encephalitis
*St. Louis encephalitis virus (SLEV)* [22,23]	mosquito (*Culex*)	bird	NA, C/SA	febrile illness, encephalitis
*West Nile virus (WNV)* [15,24,25,26,27]	mosquito (*Culex*)	bird	NA, C/SA, AF, EU, ME, AS, AU	febrile illness, encephalitis

^1^ NA = N. America, C/SA = C./S. America, AF = Africa, EU = Europe, ME = Middle East, AS = Asia, AU = Australia.

**Table 2:** Effect of temperature on vector competence for zoonotic arboviruses.

	Virus	Strain	Species	Population(s)	Temperatures (°C)	Results
**Chamberlain and Sudia, 1955**	EEEV	AR167	*Ae. triseratus*	colony	21, 27, 32, 21–32 (fluctuating)	Decreased EIP with increased temperatures. Fluctuating similar to mean temperatures
**Hurlbut 1973**	SLEV	1966	*Cx. quinque-fasciatus*	San Antonio, TX (colonized)	10–30	Decreased EIP with increased temperatures from 10–30 °C
**Richards et al., 2009**	SLEV	TBH28	*Cx. quinque-fasciatus*	Alachua/Indian River Co, FL (colonized)	25, 28	Increased viral load and competence at higher temperature (28 °C). Magnitude of effect is population, dose and age dependent.
**Takahashi 1976**	JEV	JaGAr #01	*Cx. tritaen-iorhynchus*	Japan (colonized)	20, 28	Higher replication and competence at 28 °C.
**Kramer et al., 1983**	WEEV	BFS1703	*Cx. tarsalis*	Kern Co, CA, F0/colony	18, 25, 32	Increased competence up to 32 °C to day 6. Decreased competence from 25 °C to 32 °C beyond day 6.
**Turell 1985**	RVFV	ZH501	*Cx. Pipiens Ae. taeniorhyn-chus*	Egypt (*Cx*, colonized) Vero Beach, FL (Ae., colonized)	13, 26, 33	Decreased EIP at higher temperatures up to 33 °C for both species. Increased infectivity at higher temperatures in *Cx. pipiens*.
**Turell 1989**	RVFV	ZH501	*Ae. fowleri*	Senegal (colonized)	17, 28, 17–28 (cycling)	Similar infection rates and decreased EIP at higher mean temperatures.
**Turell 1993**	RVFV VEEV	RVFV ZH501 VEEV IC-V3000	*Ae. taeniorhyn-chus*	Vero Beach, FL (colonized)	19, 26	Lower rearing temperature increased susceptibility. Higher holding temperature (26 °C) decreased EIP.
**Brubaker and Turell, 1998**	RVFV	ZH501	*Cx. pipiens*	Egypt (colonized)	13, 17, 19, 26	Increased competence (including infection) with increased temperatures up to 26 °C.
**Kay and Jennings, 2002**	RRV	B94/20	*Ae. vigilax*	Townsville, Queensland (colonized)	18, 25, 32	Similar competence among temperatures through day 7 PF. Decreased competence at day 14 PF at 32 °C.
**Reisen et al., 2006**	WNV, SLEV, WEEV	WNV NY99, WNV SA, SLEV BFS1750, WEEV BFS1703	*Cx. tarsalis*, *Cx. univitattus*	Kern Co., CA (colonized)	10, 14, 18, 22, 26, 30	Decreased EIP and increased viral load with increased temperature up to 30 °C and increased transmission rate over 18 °C. Strain and species-specific differences in magnitude of effect.
**Cornel et al., 1993**	WNV	H442	*Cx. univittatus*	Johannesburg, South Africa F1-F8	14, 18, 23.5 (cycling), 26, 30	Decreased EIP and increased replication/competence up to 26 °C (similar at 30 °C).
**Dohm et al., 2002**	WNV	NY99	*Cx. pipiens*	Westchester, NY F3-F4	18, 20, 26, or 30	Decreased EIP up to 30 °C and increased dissemination and transmission rates over 20 °C.
**Richards et al., 2007**	WNV	WN-FL03p2-3	*Cx. quinque-fasciatus*	Gainesville, FL (colonized)	25, 28, 30	Increased overall competence with increased temperatures up to 30 °C, yet dissemination rates lower at intermediated temperature (28 °C).
**Kilpatrick et al., 2008**	WNV	NY99-3356 WN02-1956	*Cx. pipiens*	PA (colonized)	15, 18, 22, 32	Increased competence with increasing temperatures up to 32 °C and magnitude of increase is virus strain-dependent.
**Anderson et al., 2010**	WNV	FL03p2-3	*Cx. quinque-fasciatus*	Gainesville, FL (colonized)	25, 28	Increased viral load and vector competence at 28 °C at high and low virus dose.
**Danforth et al., 2015**	WNV	NY99/COAV03, KERN11	*Cx. tarsalis*	Kern Co, CA (colonized)	22, 30	Decreased EIP and increased transmission rates at higher temperature (30 °C). No effect of viral strain found.
**Vogels et al., 2016**	WNV	Lin. 2, Greece 2010	*Cx. Pipiens Cx. Molestus* hybrids	Netherlands, F3-F5	18, 23, 28	Increased infection and transmission up to 28 °C for *Cx. pipiens* and hybrids. Decreased competence from 23 °C to 28 °C in *Cx. molestus*.
**Danforth et al., 2016**	WNV	KERN11	*Cx. tarsalis*	Kern Co, CA (colonized)	14.2, 21.5, 26.5, 29 (mean) 11.0, 13.5, 10.1, 14.2 (DTR)	Decreased EIP and increased transmissibility with increased temperatures. Results statistically similar to constant temperatures.
**Vogels et al., 2017**	WNV	Lin. 2, Greece 2010	*Cx. pipiens*	Netherlands, Italy, F4-F6	18, 23, 28	Increased infection and transmission from 18 °C to 23 °C in both populations and a further increase in transmission in Italian population from 23 °C to 28 °C.

**Table 3:** Literature search results that include individual viruses and temperature as keywords, 1983–2019.

Virus ^1^	# Studies Found ^2^	# Studies + Temperature	% of Research on Temperature	% of ALL Found Studies on Temperature
EEEV	608	34	6	3
WEEV	433	45	10	5
SINV	2873 ^4^	195 ^4^	7	20
RRV	843	70	8	7
BFV	178	19	11	2
VEEV	955	38	4	4
MAV	205	12	6	1
LACV	573	27	5	3
RVFV	1648	97	6	10
JEV	5426	137	3	14
MVEV	291	9	3	1
SLEV ^3^	687	49	7	5
WNV	11310	526	5	53

^1^ Virus abbreviations defined in Table 1; ^2^ literature search performed on 4 October, 2019. All others performed on 23 May, 2019; ^3^ search term: (Saint OR St) Louis Encephalitis virus; ^4^ most of the SINV results found here were excluded from the review as they focused on molecular techniques unrelated to the virus in the environment.
